# Feasibility and Challenges for Sequential Treatments in ALK-Rearranged Non-Small-Cell Lung Cancer

**DOI:** 10.3389/fonc.2021.670483

**Published:** 2021-04-20

**Authors:** Mei Elsayed, Farastuk Bozorgmehr, Daniel Kazdal, Anna-Lena Volckmar, Holger Sültmann, Jürgen R. Fischer, Mark Kriegsmann, Albrecht Stenzinger, Michael Thomas, Petros Christopoulos

**Affiliations:** ^1^ Department of Thoracic Oncology, Thoraxklinik and National Center for Tumor diseases (NCT) at Heidelberg University Hospital, Heidelberg, Germany; ^2^ Translational Lung Research Center Heidelberg, German Center for Lung Research (DZL), Heidelberg, Germany; ^3^ Institute of Pathology, Heidelberg University Hospital, Heidelberg, Germany; ^4^ Division of Cancer Genome Research, German Cancer Research Center (DKFZ), Heidelberg, Germany; ^5^ Department of Thoracic Oncology, Lungenklinik Löwenstein, Löwenstein, Germany

**Keywords:** ALK-rearranged non-small-cell lung cancer, tyrosine kinase inhibitors, chemotherapy, sequential therapies, overall survival

## Abstract

**Background:**

Anaplastic lymphoma kinase-rearranged non-small-cell lung cancer (ALK^+^ NSCLC) is a model disease for use of targeted therapies (TKI), which are administered sequentially to maximize patient survival.

**Methods:**

We retrospectively analyzed the flow of 145 consecutive TKI-treated ALK^+^ NSCLC patients across therapy lines. Suitable patients that could not receive an available next-line therapy (“attrition”) were determined separately for various treatments, based on the approval status of the respective targeted drugs when each treatment failure occurred in each patient.

**Results:**

At the time of analysis, 70/144 (49%) evaluable patients were still alive. Attrition rates related to targeted treatments were approximately 25-30% and similar for administration of a second-generation (2G) ALK inhibitor (22%, 17/79) or any subsequent systemic therapy (27%, 27/96) after crizotinib, and for the administration of lorlatinib (27%, 6/22) or any subsequent systemic therapy (25%, 15/61) after any 2G TKI. The rate of chemotherapy implementation was 67% (62/93). Both administration of additional TKI (median overall survival [mOS] 59 *vs.* 41 months for multiple *vs.* one TKI lines, logrank p=0.002), and chemotherapy (mOS 41 vs. 16 months, logrank p<0.001) were significantly associated with longer survival. Main reason for patients foregoing any subsequent systemic treatment was rapid clinical deterioration (n=40/43 or 93%) caused by tumor progression. In 2/3 of cases (29/43), death occurred under the first failing therapy, while in 11/43 the treatment was switched, but the patient did not respond, deteriorated further, and died within 8 weeks.

**Conclusions:**

Despite absence of regulatory obstacles and no requirement for specific acquired mutations, 25-30% of ALK^+^ NSCLC patients forego subsequent systemic therapy due to rapid clinical deterioration, in several cases (approximately 1/3) associated with an ineffective first next-line choice. These results underline the need for closer patient monitoring and broader profiling in order to support earlier and better directed use of available therapies.

## Introduction

Anaplastic lymphoma kinase-rearranged (ALK^+^) non-small-cell lung cancer (NSCLC) is a model disease for the implementation of targeted therapies in thoracic oncology (1). The first-generation (1G) tyrosine kinase inhibitor (TKI) crizotinib was approved by the European Medicines Agency (EMA) already in August 2011, based on superior efficacy and tolerability compared to conventional chemotherapy (2). During the last two years, it was superseded by second-generation (2G) compounds in the upfront setting, especially alectinib and brigatinib, whose even better systemic and intracranial activity was reflected in longer-lasting responses and a median overall survival (OS) exceeding 5 years in the ALEX trial (3, 4). More recently, the third-generation (3G) drug lorlatinib demonstrated even higher efficacy and is currently the standard option after failure of any next-generation ALK TKI (5, 6). Accumulating evidence from real-world retrospective analyses as well as clinical trials underlines the importance of sequential TKI administration in order to optimize patient outcome (3, 7, 8). Indeed, newer compounds are more potent ALK inhibitors and show broader activity against *ALK* resistance mutations, therefore they can salvage patients failing older TKI (9). At the same time, a current characteristic of ALK^+^ disease is that the approval of targeted pharmaceuticals for next-line administration is “open”, i.e. does not depend on the results of molecular retesting and presence of any specific resistance mutation, in contrast, for example, to the administration of osimertinib after failure of 1G/2G EGFR inhibitors, which requires detection of *EGFR* T790M (10). Nevertheless, in clinical practice a considerable number of ALK^+^ patients will forego subsequent therapy. The aim of this study is to provide an accurate estimate for the frequency and causes of this problem.

## Patients and Methods

### Study Population and Endpoints

This retrospective study included all consecutive ALK^+^ NSCLC patients treated in the Thoraxklinik Heidelberg and Lungenklinik Löwenstein from 2011 until 2020. In order to provide a detailed and accurate picture of sequential treatments for ALK^+^ NSCLC and their impact on patient outcome, study endpoints considered each relevant pharmaceutical class and each therapeutic context separately: i) administration of 2G ALK inhibitors after crizotinib (1G); ii) administration of lorlatinib (3G) after 2G compounds; iii) administration of any treatment after crizotinib (1G); iv) administration of any treatment after 2G compounds; v) administration of chemotherapy at any time during treatment; vi) OS after administration of multiple TKI *vs.* one single TKI line; vii) OS with TKI-only treatment *vs.* treatment with both TKI and chemotherapy. Deceased patients were considered to have been candidates for a specific class of next-line targeted therapy, when their date of death was after the time of the earliest approval within this TKI class by the European Medicines Agency (EMA), which was May 2015 for 2G ALK inhibitors (ceritinib was approved first in May 2015, followed by alectinib in February 2017 and brigatinib in November 2018), and May 2019 for lorlatinib. In addition, all patients were considered potentially eligible for chemotherapy, since the baseline ECOG performance status (PS) in our cohort ranged from 0-2, and therefore every patient could have received at least some mild cytotoxic treatment.

### Data Collection and Statistical Analysis

Histologic diagnosis of NSCLC and detection of *ALK* gene fusions were performed at the Institute of Pathology Heidelberg on tissue specimens according to the criteria of the current WHO Classification (2015) for lung cancer (11). Newly diagnosed cases were screened for the presence of an ALK alteration by fluorescence *in situ* hybridization (FISH, ZytoLight SPEC ALK probe, ZytoVision GmbH, Bremerhaven, Germany) and reverse-transcription polymerase-chain reaction (RT-PCR) until 2015, or by immunohistochemistry (IHC, D5F3 clone, Roche, Mannheim, Germany) and RNA-based next-generation sequencing (NGS, ThermoFisher Lung Cancer Fusion Panel, Waltham, MA, USA) thereafter, as previously described (12). Clinical data were systematically collected from the medical records, including review of the patients’ radiological images, i.e. chest/abdomen CT and brain MRI-based restaging every 6-12 weeks, by the investigators. For deceased patients, both given treatments and missed treatments were considered. For patients still under therapy, only given treatments were considered, since these patients could still receive or not receive some additional treatment in the further course. For every single patient who was eligible for some type of subsequent treatment, i.e. 2G TKI after crizotinib, lorlatinib after 2G ALK inhibitors, or chemotherapy after any TKI, but did not receive it until death, the clinical course as documented in the records was analyzed to understand why the treatment was missed. The relationship between survival and sequential administration of TKI was analyzed in the entire patient population. For chemotherapy, the survival analysis was performed in the subset of deceased patients, for which the entire disease trajectory could be analyzed, because chemotherapy is generally given after targeted therapies in ALK^+^ NSCLC. Survival data were analyzed according to Kaplan-Meier and compared between patient groups with the logrank test. Follow-up time was calculated using the reverse Kaplan-Meier method (13). Categorical data were compared using a chi-square test, while 95% confidence intervals (CI) of proportions were calculated with the modified Wald method (14). Statistical calculations were performed with SPSS version 24 (IBM, NY, USA) and GraphPad Prism version 9 (La Jolla, CA, USA), which was also used to make the plots.

### Ethics

This study was approved by the ethics committee of the Heidelberg University (S-145/2017 and S-469/2017). Since this was a non-interventional, retrospective study, informed consent was obtained whenever possible, but its need for every participant was waived by the ethics committee.

## Results

### Evaluable Study Patients

**Figure 1:**
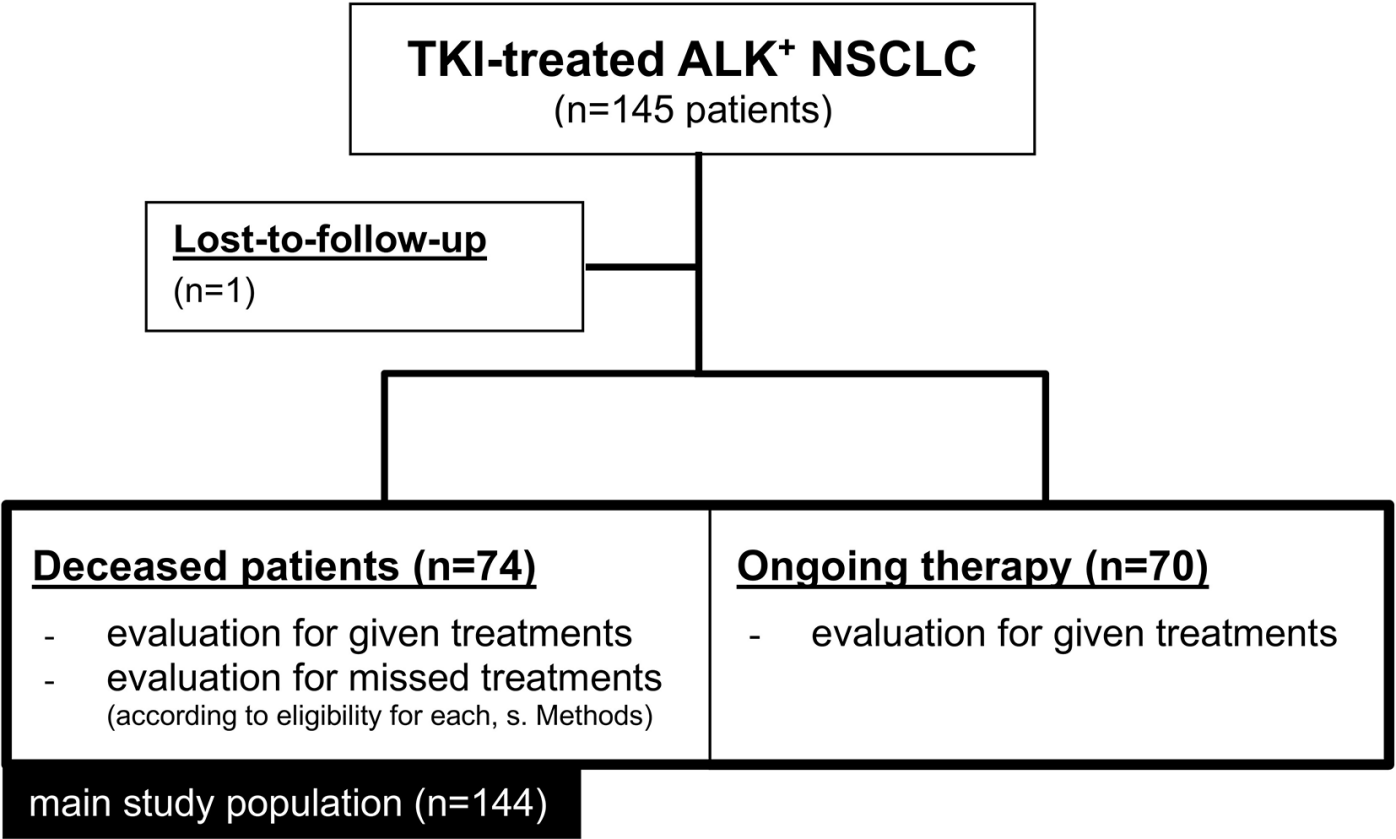
Flowchart of study patients.

Overall, 145 eligible ALK^+^ NSCLC patients that had received at least one ALK inhibitor were identified, of which 144 had complete follow-up data and were included in this study (**Figure 1**). Their clinical characteristics are given in **Table 1**. Median age was 57 years, while the majority were female (60%), never-light smokers (77%) with an ECOG performance status at initial diagnosis of 0-1. The median number of treatment lines was 2 (range 1-9), the median overall survival 51 months (CI 44-59), and the median follow-up 54 months (95% CI 46-61, **Table 1**). At the time of data cut-off, 70 patients were still alive, while 74 patients had died.

**Table 1:** Characteristics of study patients.

		All study patients (n = 144)	Deceased patients (n = 74)
Age, median (SD; range)		57 (14; 21-85)	57 (15; 21-85)
Sex, % female (n)		60 (86)	57 (42)
ECOG, % (n)	PS 0-1	97 (140)	95 (70)
	PS 2	3 (54)	5 (4)
Never/light-smokers (< 10 pack-years), % (n)		77 (105)	78 (53)
*ALK* variant, % (n)	*EML-ALK* V3 (E6;A20)	41 (51)	50 (32)
	*EML-ALK* V1/V2	49 (60)	39 (25)
	other^1^	10 (11)	11 (7)
*TP53* status at diagnosis	mutated^2^	21 (24)	25 (15)
TKI lines, median (SD; range)		1 (1.1; 1-5)	1 (1.1; 1-5)
All treatment lines, median (SD; range)		2 (1.9; 1-9)	3 (1.8; 1-9)
OS months, median (95% CI)		51 (44-59)	27 (20-33)
Follow-up months, median (95% CI)		54 (46-61)	26 (20-33)

ECOG PS, ECOG performance status; OS, overall survival; SD, standard deviation; TKI, tyrosine kinase inhibitor; 95% CI, 95% confidence interval.

^1^the ALK variant could be typed for 122 cases; other patients had E18:A20 (n=4), HIP1-ALK (n=2), KCL-ALK (n=4), KIFB-ALK (n=1).

^2^the TP53 status could be determined for 117 cases.

### Rate of Sequential Treatments and Impact on Patient Survival

**Figure 2:**
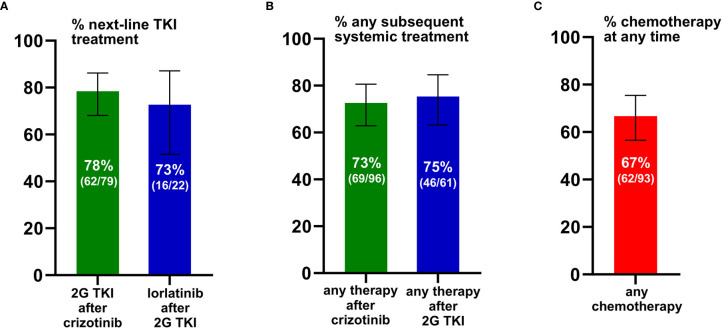
Feasibility of sequential therapies in ALK^+^ NSCLC. **(A)** Any second-generation (2G) ALK inhibitor was given to 78% (62/79) of eligible patients failing crizotinib, while lorlatinib (3G) was given to 73% (16/22) eligible patients failing any 2G ALK inhibitor. For each analysis, the reference population of eligible patients included all those who could have received the respective subsequent drug, based on approval by the EMA at the time of the patients’ treatment failure, as explained in the *Methods*. Error bars indicate 95% confidence intervals. **(B)** Any systemic anticancer treatment (i.e. any ALK inhibitor or chemotherapy) was given to 73% (69/96) patients failing crizotinib, and to 75% (46/61) patients failing 2G ALK inhibitors. Error bars indicate 95% confidence intervals (CI). **(C)** Chemotherapy was given to 67% (62/93) of patients. Error bars indicate the 95% confidence interval.

**Figure 3:**
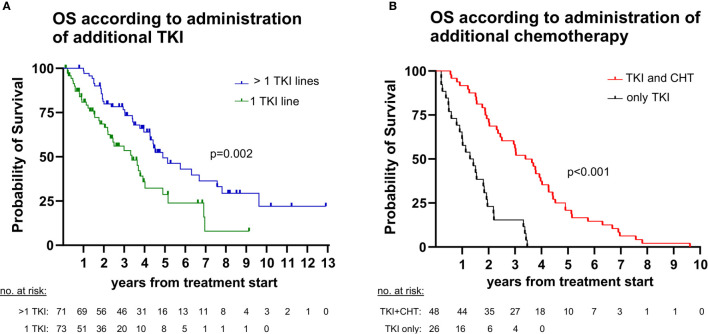
Impact of sequential therapies on overall survival (OS) in ALK^+^ NSCLC. **(A)** The median OS from start of treatment for metastatic disease was 59 months (95% confidence interval [CI] 43-74) for patients with > 1 tyrosine kinase inhibitor (TKI) lines *vs.* 41 months (CI 26-55) for patients with a single TKI line (logrank p=0.002). **(B)** The median OS from start of treatment for metastatic disease was 41 months (CI 30-51) for patients who also received chemotherapy (CHT) in addition to TKI *vs.* 16 months (CI 8-23) for patients who were treated with TKI only (logrank <0.001). Since chemotherapy is generally administered after TKI for ALK^+^ NSCLC, the analysis regarding chemotherapy was performed in the subset of deceased patients (n=74, **Figure 1**), for which the entire disease trajectory could be studied.

We first analyzed the percentage of ALK^+^ NSCLC patients who received next-generation ALK inhibitors after failure of 1G or 2G TKI. The reference population for each of these calculations were all patients that could have received the respective drugs, based on approval by the EMA before the time of the patients’ treatment failure (“eligible patients”), as explained in the Methods and shown in **Figure 2**. 2G TKI were offered to 78% (62/79, CI 68-86%) of eligible patients after crizotinib failure, while lorlatinib was offered to 73% (16/22, CI 52-87%) of eligible patients failing 2G ALK inhibitors (**Figure 2A**). Among patients failing crizotinib, any subsequent anticancer treatment (including chemotherapy) was given to 73% (69/96, CI 63-81%) of patients, while among patients failing 2G ALK inhibitors any subsequent anticancer treatment was given to 75% of patients (46/61, CI 63-85%, **Figure 2B**). Chemotherapy at any time during treatment was given to 67% (62/93, CI 57-75%) of patients (**Figure 2C**). Both the administration of additional TKI and additional chemotherapy were significantly associated with longer survival of TKI-treated ALK^+^ NSCLC patients (**Figure 3**): the median OS from start of treatment for metastatic disease was 59 months (CI 43-74) for patients who received multiple TKI lines *vs.* 41 months (CI 26-55) for patients who received a single TKI line only (logrank p=0.002, **Figure 3A**); while the median OS was 41 months (CI 30-51) for patients who also received chemotherapy in addition to TKI, *vs.* 16 months (CI 8-23) for patients who received TKI only, but no chemotherapy (logrank p<0.001, **Figure 3B**).

### Analysis of Clinical Courses for Patients Foregoing Subsequent Treatment

**Figure 4:**
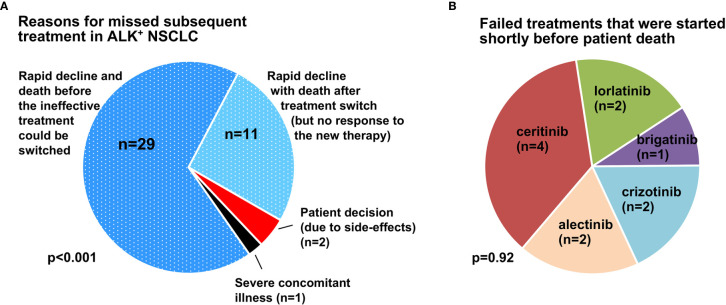
Main causes for missed subsequent treatment in ALK ^+^ NSCLC. **(A)** The main reason for missed subsequent treatment in ALK^+^ NSCLC patients was clinical deterioration due to rapid disease progression (n=40/43), while patient decision against further therapy (2/43, both due to treatment side-effects) and severe concomitant illness (n=1/43, one patient with advanced chronic obstructive pulmonary disease and worsening respiratory failure) were rare. In two-thirds of cases (29/43 or 67%), the patient died while on the first failing therapy, while in approximately 25% (11/43), the treatment was switched, but the patient did not respond, deteriorated further, and died within 8 weeks. The p-value was derived by chi-square testing across the various categories. **(B)** ALK TKI used in deteriorating cases as ineffective salvage therapy shortly before death (≤ 8 weeks). The p-value was derived by chi-square testing across the various categories.

For all patients who missed subsequent treatment (2G ALK TKI, or 3G ALK TKI, or chemotherapy) as shown in **Figure 2**, we performed a detailed examination of their clinical courses as documented in the records in order to gain insight into the underlying circumstances. This showed that the main reason for ALK^+^ NSCLC patients missing subsequent treatments, either TKI or chemotherapy, was rapid clinical deterioration (n=40/43 or 93%, **Figure 4**). In two-thirds of cases (29/43 or 67%), the patient died while on the first failing therapy, whereas in approximately 25% (11/43), the treatment was switched, but the patient did not respond, deteriorated further and died within 8 weeks. Other causes, such as patient decision against further anticancer therapy (n=2, both due to severe TKI side effects) and severe concomitant illness (n=1, with advanced chronic obstructive pulmonary disease and progressive respiratory failure under therapy) were rare.

## Discussion

Sequential administration of effective drugs is critical in order to maximize therapeutic benefit and the survival of patients with metastatic lung cancer (15). In the special case of ALK^+^ NSCLC, next-line use of ALK inhibitors is “open”, i.e. not dependent on the molecular results of a tumor rebiopsy at the time of disease progression. Due to this lack of regulatory obstacles in ALK^+^ NSCLC, main focus of previous studies has been the efficacy rather than the feasibility of therapeutic sequencing (8, 16–19). Systematic analysis of the latter is endowed with serious difficulties, mainly the rarity and generally better prognosis of the disease compared to EGFR^+^ and wild-type NSCLC (7), which necessitate longer study intervals in order to recruit sufficient patient numbers, the multitude of ALK-directed compounds, which are used in variable order, as well as the rapidly changing landscape of regulatory approval, which influences the availability and prioritization of various drugs.

Using a large, homogenous patient population, considering each therapeutic context separately, and taking into account the longitudinal availability of various ALK inhibitors for each patient, our study shows that the attrition of ALK^+^ NSCLC patients between different treatments is approximately 25-30% (**Figure 2**). This holds true for the administration of 2G compounds or any treatment after crizotinib, as well as for the administration of lorlatinib or any treatment after 2G TKI. The slightly higher (33%) attrition observed for additional chemotherapy is probably due to its worse efficacy and tolerability compared to TKI, which render it less desirable early in the disease course, and also less suitable for heavily pretreated patients (2, 20, 21). Interestingly, both percentages are considerably lower than the approximately 50% loss observed between first- and second-line palliative chemotherapy in metastatic NSCLC (22–25), but comparable to the approximately 30% loss reported after first-line TKI treatment in EGFR^+^ NSCLC patients, both in the standard arm of the phase 3 FLAURA trial [(32%, Supplementary Table S2 of the respective original publication (26)] as well as in real-world analyses from certified German lung cancer centers, including own data (27–29). To our knowledge, so far, no other detailed estimates of attrition according to each failing treatment and next-line option exist in the literature for ALK^+^ NSCLC. Of note, the long timespan of our cohort from 2011-2020 is an important advantage, because it permits more balanced capturing of favorable and unfavorable cases. In contrast, attrition rates based on interim results of prospective clinical trials are likely to be enriched for cases with worse outcome and a higher likelihood to miss subsequent treatment, since they are necessarily focused on the patient subset with earlier treatment failure and/or death. For example, in the recently published update of the ALEX trial, the percentage of patients receiving any treatment after alectinib or crizotinib was approximately 60% (3), i.e. somewhat lower than that observed in the present study (70-75%, **Figure 2**).

This association between shorter survival and lack of subsequent therapy was evident in our patients regarding both TKI and chemotherapy (**Figure 3**). Main cause for most cases (67% or 29/43) was rapid clinical deterioration with death before any salvage therapy could be initiated (**Figure 4A**). The overall percentage of patients lost under treatment with ALK inhibitors in this “direct” way, i.e. 25-30% (**Figure 2**) x 67% (**Figure 4A**, dark blue sector) ≈ 20% defines the theoretical upper limit, i.e. approximately 80%, for implementation of any subsequent therapies in ALK^+^ NSCLC. This limit will acquire greater importance in the near future, because it is expected to equally hinder feasibility of all next-line targeted therapies for ALK^+^ NSCLC, for example also newly-developed fourth-generation ALK inhibitors directed against compound ALK mutations (30), or other drugs targeting other actionable resistance mechanisms, such as acquired *MET* amplifications or *KRAS* mutations (9). Therefore, this 20% direct patient loss between lines represents currently an important argument for closer patient monitoring, in order to achieve earlier detection of treatment failure, so that subsequent therapies can be selected and started while the patient can still benefit from them. Besides, in approximately 25-30% (**Figure 2**) x 25% (**Figure 4A**, light blue sector) ≈ 5-10% of cases, various next-line TKI were started, but no response occurred, further deterioration followed, and the patients died within a few weeks (**Figure 4B**). This “secondary” patient loss highlights the additional need for improved molecular profiling of acquired resistance in order to support *a priori* selection of effective drugs for subsequent treatment, since there might be no second chance. Regarding both needs, i.e. for earlier detection and for improved profiling of TKI failure, a very promising approach are longitudinal liquid biopsies (circulating tumor [ct]DNA assays). These can not only identify acquired *ALK* resistance mutations and other actionable alterations, but also monitor the tumor remission status and emergence of high-risk features, for example acquisition of *TP53* mutations (31), in a minimally-invasive manner (32, 33). Important practical advantages of blood ctDNA assays for newly symptomatic and/or clinically deteriorating patients are the easier sample collection and earlier availability of results compared to percutaneous or bronchoscopic tissue biopsies (34). For many cases without detectable alterations of individual genes, the trimmed median absolute deviation from copy number neutrality (t-MAD score) determined using low-coverage (0.5-1x) whole genome sequencing, has recently demonstrated potential clinical utility as an alternative monitoring parameter (35). Of note, monitoring of electronic patient−reported outcomes (ePROs) under chemotherapy for various solid tumors was associated with significantly longer survival in a pivotal study, and could therefore represent a cost-efficient alternative method to improve care of ALK^+^ NSCLC patients, since quality of life can fluctuate under treatment with TKI, as well (36, 37).

With the advent of highly potent TKI, the importance of chemotherapy for ALK^+^ NSCLC has diminished, but should not be neglected, as it confers an additional survival benefit (**Figure 3B**). In clinical practice, a particular challenge is the optimal timing of switch from TKI to chemotherapy: neither too early, in order to maximize chemotherapy-free time, nor too late, after the patient is not fit enough for cytotoxics anymore. Even though the association between administration of chemotherapy or any subsequent therapy and longer survival observed in our study could be partly indirect, i.e. due to the better clinical condition of the respective patients, this does not lessen the importance of improved monitoring and profiling in order to preserve patient fitness and facilitate therapeutic sequencing (21). In fact, among the 11 cases in our series, who received various TKI as salvage therapies, but did not respond and died soon thereafter (**Figure 4B**), some might have benefited more from an earlier decision for chemotherapy, instead. Systematic use of tissue and liquid rebiopsies at the time of disease progression in the future could help identify patients with off-target resistance mechanisms, for which further treatment with ALK inhibitors has low chances of success, and thus indirectly support timely decisions for alternative targeted drugs or chemotherapy (1, 9, 21).

## Conclusion

In summary, despite lack of regulatory obstacles, the attrition of ALK^+^ NSCLC patients between various treatment lines is approximately 25% and represents an important limitation for survival. Main problem is rapid clinical deterioration caused by tumor progression, which could be counteracted in future studies through closer radiologic and/or ctDNA monitoring and broader molecular profiling.

## Data Availability Statement

The data supporting the results of this study will be made available by the corresponding author upon reasonable request.

## Ethics Statement

The studies involving human participants were reviewed and approved by ethics committee of Heidelberg University (S-145/2017 and S-469/2017). Written informed consent for participation was not required for this study in accordance with the national legislation and the institutional rules.

## Author Contributions

ME: conceptualization, methodology, investigation, data curation, formal analysis, visualization, and writing - original draft. FB: investigation, data curation, validation, and writing - review & editing. DK: investigation, data curation, validation, and writing – original draft. A-LV: investigation, data curation, validation, and writing - review & editing. HS: conceptualization, validation, supervision, and writing - review & editing. JF: investigation, data curation, validation, writing - review & editing. MK: investigation, data curation, and writing - review & editing. AS: validation, supervision, project administration, and writing - review & editing. MT: conceptualization, methodology, validation, supervision, funding acquisition, and writing - review & editing. PC: conceptualization, methodology, investigation, data curation, formal analysis, visualization, supervision, project administration, writing - original draft, and writing - review & editing. All authors contributed to the article and approved the submitted version.

## Funding

This work was supported by the German Center for Lung Research (DZL).

## Conflict of Interest

FB: research funding from BMS and travel grants from BMS and MSD. DK: advisory board and speaker’s honoraria from AstraZeneca, BMS, Pfizer. HS: advisory board and speaker’s honoraria from Roche. JF: advisory board honoraria from Boehringer, Roche, Celgene and AstraZeneca. AS: advisory board honoraria from BMS, AstraZeneca, ThermoFisher, Novartis, speaker’s honoraria from BMS, Illumina, AstraZeneca, Novartis, ThermoFisher, MSD, Roche, and research funding from Chugai. MT: advisory board honoraria from Novartis, Lilly, BMS, MSD, Roche, Celgene, Takeda, AbbVie, Boehringer, speaker’s honoraria from Lilly, MSD, Takeda, research funding from AstraZeneca, BMS, Celgene, Novartis, Roche and travel grants from BMS, MSD, Novartis, Boehringer. PC: research funding from AstraZeneca, Novartis, Roche, Takeda, and advisory board/lecture fees from AstraZeneca, Boehringer Ingelheim, Chugai, Novartis, Pfizer, Roche, Takeda.

The remaining authors declare that the research was conducted in the absence of any commercial or financial relationships that could be construed as a potential conflict of interest.
